# An exploratory study to estimate cost-effectiveness threshold value for life saving treatments in western Iran

**DOI:** 10.1186/s12962-020-00241-9

**Published:** 2020-10-23

**Authors:** Najmeh Moradi, Abraha Woldemichael, Parisa Malekian, Delnia Moradi Rotvandi, Satar Rezaei

**Affiliations:** 1 Health Management and Economics Research Center, Iran University of Medical Sciences, Tehran, Iran; 2 Department of Health Systems, School of Public Health, College of Health Sciences, Mekelle University, Mekelle, Ethiopia; 3 Students Researches Committee, Kermanshah University of Medical Sciences, Kermanshah, Iran; 4 Research Center for Environmental Determinants of Health, Health Institute, Kermanshah University of Medical Sciences, Kermanshah, Iran

**Keywords:** Cost-effectiveness, Life-saving treatment, Willingness to pay, Quality-adjusted life year, Iran

## Abstract

**Background:**

Cost-effectiveness analysis provides a crucial means for evidence-informed decision-making on resource allocation. This study aims to elicit individuals' willingness to pay (WTP) for one additional quality-adjusted life-year (QALY) gained from life-saving treatment and associated factors in Kermanshah city, western Iran.

**Methods:**

We conducted a cross-sectional study on a total of 847 adults aged 18 years and above to elicit their WTP for one additional QALY gained by oneself and a family member using a hypothetical life-saving treatment. We used a multistage sampling technique to select the samples, and the Iranian version of EQ-5D-3L, and visual analogue scale (VAS) measures to obtain the participants’ health utility value. The Tobit regression model was used to identify the factors affecting WTP per QALY values.

**Results:**

The mean WTP value and standard deviation (SD) was US$ 862 (3,224) for the respondents. The mean utility values using EQ-5D-3L and VAS methods for respondents were 0.779 and 0.800, respectively. Besides, the WTP for the additional QALY gained by the individual participants using the EQ-5D-3L and VAS methods were respectively US$ 1,202 and US$ 1,101, while the estimated value of the family members was US$ 1,355 (SD = 3,993). The Tobit regression models indicated that monthly income, education level, sex, and birthplace were statistically significantly associated (p < 0.05) with both the WTP for the extra QALY values using the EQ-5D-3L and the VAS methods. Educational level and monthly income also showed statistically significant relationships with the WTP for the additional QALY gained by the family members (p < 0.05).

**Conclusion:**

Our findings indicated that the participants' WTP value of the additional QALY gained from the hypothetical life-saving treatment was in the range of 0.20–0.24 of the gross domestic product (GDP) per capita of Iran. This value is far lower than the World Health Organization (WHO) recommended CE threshold value of one. This wide gap reflects the challenges the health system is facing and requires further research for defining the most appropriate CE threshold at the local level.

## Background

The scarcity of healthcare resources and the increasing clients’ treatment demands challenge the decisions on resource allocation such as financial reimbursements, especially in the health systems of resource-constrained countries [1, 2]. The incremental cost-effectiveness ratio (ICER) can be applied to compare the costs and health gains from two or more alternative interventions and is a widely used to handle challenging decisions [3–5]. The quality-adjusted life years (QALY), which includes both the quality and quantity of life, is one of the commonly used health outcome indicators, and the cost per QALY is a ratio of the additional cost per QALY gained [6, 7]. One QALY equates to one year in perfect health. An intervention is considered cost-effective [7] if the ICER value lies below an established threshold value, and vice versa [5, 8]. Despite this commonly used approach, there is no single standard to estimate the CE threshold [9]. The World Health Organization (WHO) considers that if the incremental cost to incremental QALYs gained ratio is less than one, or a value of one to three times the per capita GDP as cost-effective intervention, with the higher value unacceptable [10, 11].

A recent study that estimated the CE threshold indicated the WHO recommended threshold value is considerably higher for the low-middle income countries (LMICs) [12]. Because of the limitation of the WHO recommended estimation, others applied the willingness to pay (WTP) method for a preferred attribute to estimate the CE threshold value [5, 13–15]. Despite methodological issues related to the use of CE threshold value approaches, there is a consensus that the different thresholds should be well defined and used for different situations such as with quality of life-improving or life-threatening conditions. Evidence indicates a higher WTP values for life-saving interventions than for quality of life-improving ones [16]. Others also reported different QALY values for different health status, and those with worse health status had a higher value than those with better health status [13].

In Iran’s health system, there seems to be a consensus in using a regional CE threshold to maximize health and efficiency. Some of the efforts demonstrating the use of the CE threshold are in the establishment of the Health Technology Analysis Office at the Ministry of Health and Medical Education (MoHME) of Iran in 2007, and; in the provision of training in the fields of health economics, health technology assessment, and pharmaco-economics in medical universities. Other efforts include the application of pharmaco-economic evaluation guidelines to include new drugs in the national drug list of the Food and Drug Administration of Iran, and the conduct of studies on economic evaluations concerning medical equipment and treatment are additional national-level efforts [17–19].

Despite the absence of clear valid criteria for the CE threshold to aid in the decision on health resources allocation, the decision-makers in Iran are implicitly applying the WHO recommendation of choosing the CE intervention(s) which has its limitations [2]. Besides, the use of the WHO criteria on the decisions made concerning the health insurance organizations’ benefits such as inclusion and exclusion healthcare is unclear. However, a study in Tehran reported an average WTP per QALY of the participants ranging from US$ 1,032 to US$ 2,666. These minimum and maximum values accounted for 0.22 to 0.56 GDP per capita of Iran in 2014 [2]. Nevertheless, to the best of our knowledge, little information is available on the monetary value of life-saving treatments and associated factors in Iran. Thus, to fill this information gap, this research strove to answer the following three main questions: What is the maximum WTP of a participant for one additional QALY from a hypothetical life-saving treatment? What is the maximum WTP of a participant if the gain in one additional QALY is for a family member? Finally, are these values associated with the explanatory variables included in the study? Eliciting the monetary value of the QALY as the threshold in CE analysis can provide useful information for evidence-informed decisions in resource allocation in Iran, and perhaps in other similar contexts.

## Methods and materials

### Setting

Kermanshah city, the capital of Kermanshah province, is located in western Iran. Based on the 2016 population census of Iran, the city had a total population of about two million people. The socio-economic status of the people is low, and the city's contribution to the national gross domestic product (GDP) is only about 1.7% to 2%.

### Study design, study period, and sample size

A cross-sectional study was conducted on a total sample of 943 adults aged 18 years and above, from the general population of Kermanshah city, to elicit their WTP for one additional QALY gained from a hypothetical life-saving treatment during September to December 2019. The Mitchell and Carson [20] was used to determine the appropriate sample size.

$$n={\left[\frac{{Z}_{1-\frac{\alpha }{2}} V}{\Delta }\right]}^{2}$$

where n represents the calculated sample size at $\alpha$=10%, V = 2.5, and $\Delta =0.1$ (the difference between the true and estimated WTP values). Thus, the computed sample size was at 786. However, considering the attrition rate of 20% and generalizability of the findings, the final sample for the study was 943. We used a multistage sampling technique to select the study participants by dividing the city into the western, eastern, central, northern, and southern geographic areas. Then, we equally divided the final sample into five (n = 189) and selected the study participants using a systematic random sampling technique.

### Data collection and variables

We used a self-administrated questionnaire to obtain data for eliciting the participants’ WTP for one additional QALY gained by oneself and a family member using a hypothetical life-saving treatment [4]. The self-administered questionnaire focused on the participants’ current health state, WTP for one additional QALY gained from life-saving treatment, and sociodemographic characteristics (Additional file 1: Appendix 1). Before the final data collection, five health economists checked the questionnaire for its content validity, a revision was made based on their opinions, and a pilot test was conducted on 30 participants to ensure the understandability of the questions and the hypothetical scenarios.

We used the Iranian version of the EQ-5D-3L as well as a visual analogue scale (VAS) measure to obtain the respondents’ health utility values [4]. The EQ-5D-3L had five dimensions consisting of mobility, self-care, usual activities, pain/discomfort, and anxiety/depression dimensions with; three-level responses of: no problems, some problems, and extreme problems. A participant had to use one of these responses in each dimension to indicate his/her current health state [21]. Again, we allowed the respondents to identify their current health state on a 100-unit thermometer analogue scale extending from 0 (almost dead) to 100 (perfect health) for the VAS valuation [4, 22].

As in a previous study [4], we used two hypothetical life-threatening condition for the individual participants and their family members to estimate their maximum WTP value for one additional QALY gained. The assumption used for the participant in the first scenario was as follows: *“Suppose you had a life-threatening disease for the past year. There is a cure (treatment) for the disease., and if you do not get the treatment now, you will die today. If you get treated, you will be back to your original health state and live only for one more year”*. In the second scenario, we presented the respondents with a similar to the first one but asked them to imagine the situation for his/her family members (a different perspective).

We elicited the maximum WTP value for one more QALY gained by a family member from the hypothetical intervention using the contingent valuation method (CVM) [23]. The CVM is one of the most commonly employed methods to elicit the WTP of individuals for one additional QALY gained using an intervention. For example, a systematic review that determined the WTP per QALY reported that 92.85% of the studies applied the CVM, and only one study used a discrete choice experiment method to estimate the WTP of participants [16]. Furthermore, we used the payment card (PC) method, one of the CVM methods, accompanied by a follow-up of open-ended questions to identify the participants’ WTP. The PC applies a visual scale consisting of a range of potential bid values presented to the respondents to indicate their best WTP value [24]. The PC was comprised of 15 bid values ranging from the lowest US$ 78 to the highest US$ 19,381, and we presented the bid to those that showed a positive attitude toward the WTP. However, we included the values below US$ 78 and above US$ 19,381 on the PC scale to avoid limiting the participants’ chance of not responding.

The follow-up questions elicited the respondents’ exact WTP values. We used values ranging from zero to more than US$ 19,380 from a pilot study conducted in 2019. In this study, we utilized the PC with closed-ended questions because it covered a wide range of bids and helps avoid fatigue and confusion of the respondents in the valuations. These limitations are likely to occur when using other CVM methods such as the dichotomous choice, bidding game formats, and the multiple bounded discrete choice methods, where the respondents had to bargain to show the WTP values. The value of US$ 1 at the time of the study was equivalent to 128,986 Iranian Rials (IRRs) [25]. Sociodemographic related variables included in the analysis were age, sex, marital status, individual monthly income, education status, health insurance coverage, birthplace, and having a chronic disease.

### Data analysis

We calculated the utility scores from the additional QALY gained using the EQ-5D-3L and VAS valuation methods. The data were initially scaled on the VAS from the best to the worst imaginable health state and then rescaling the scores of the respondents from 100 to 0 using the following formula:

$$ {\text{VAS}}_{{\text{rchs }}} = \frac{{{\text{VAS}}_{{{\text{raw}}}} - {\text{Dead}}_{{{\text{raw}}}} }}{{^{\prime}11111^{\prime}_{{\text{raw }}} - {\text{Dead}}_{{{\text{raw}}}} }} $$

where VAS_rchs_ and VAS_raw_ respectively represent the scores of the rescaled current health state and current health state. Death_raw_ and ′11111′_raw_ are the scores of almost dead and perfect health states, respectively.

In this study, the additional QALY gained by each respondent was the difference between the utility measure of the current health state using the EQ-5D-3L or VAS and the almost dead health state, and calculated using the following mathematical equation:

$$\mathrm{WTP for one additional QALY gained}=\frac{\mathrm{WTP value}}{{\mathrm{U}}_{\mathrm{chs}} -{\mathrm{ U}}_{\mathrm{death}}}$$

where U_death_ is the utility from the dead health state which is equal to 0.000 and U_chs_ is the utility from the current health state. The WTP for the additional QALY gained is the amount of WTP per an additional QALY gained by oneself or a family member.

We used the Mann–Whitney and chi-square tests to explore the association between the continuous and categorical explanatory variables and the WTP for the life-saving treatment of the respondents, respectively. The data on WTP for the additional QALY gained from the life-saving treatment using the EQ-5D-3L and VAS methods were positively skewed. Similar to previous studies [26–29], we applied the Tobit regression model to explore the relationship between the WTP for the additional QALY gained and the explanatory variables, and to handle the possible limitations that may arise when using other models. Furthermore, we estimated the marginal effect of the $${\beta }^{*}$$ and $${\beta }^{**}$$, where $${\beta }^{*}$$ is the explained marginal effect for the probability of being uncensored and $${\beta }^{**}$$ is the explained marginal effect for the expected WTP value conditional on being uncensored: E (WTP|WTP > 0). The age, gender, educational level, health insurance coverage, marital status, birthplace, chronic disease status on oneself, chronic disease status in a family member, death of a family member in the past year, and monthly household income were the dependent variables. The Stata statistical software package version 14.2 performed all the analyses, and we considered the findings as statistically significant at the p-value of less than 0.05.

## Results

A total sample of 847 adult Iranians aged 18 years and above living in Kermanshah city participated in the study. The mean age of the participants was 33.6 years, with a standard deviation (± SD) of 12.1 years, and male and female participants accounted for 45.4% and 54.6%, respectively (Table 1). One-hundred and forty-eight of the respondents (17.5%) had a monthly income of less than US$ 78, while 158 (18.6%) had more than US$ 310 (US$ 1 = IRR 128,986). Nearly 19% of the respondents had chronic diseases, 15.2% had a history of the death of a family member in the last year, and around two-thirds (65%) were willing to pay for the hypothetical life-saving treatment for themselves. The univariate analysis indicated that gender, educational status, health insurance coverage, birthplace, and monthly income were statistically significantly associated with the WTP for the life-saving treatment.

**Table 1:** Frequency distribution, Mann–Whitney and chi-square analysis of willingness to pay for life-saving treatment

Variable	Willing to pay (n = 551)	Not willing to pay (n = 296)	n(%) or mean (± SD)	p value
Age, in year	32.9	34.7	33.6 (12.1)	0.130
Sex				
Male	233 (42.3%)	152 (51.3%)	385 (45.4%)	
Female	318 (57.7%)	144 (48.6%)	462 (54.6%)	0.012**
Marital status				
Married	121 (40.9%)	235 (42.6%)	356 (42.0%)	
Single	162 (54.7)	294 (53.4%)	456 (53.8%)	
Others	13 (4.4)	22 (4.0%)	35 (4.2%)	0.867
Education status				
Illiterate	26 (4.7%)	21 (7.1%)	47 (5.5%)	
Primary and secondary school	133 (24.1%)	87 (29.4%)	220 (26.0%)	
Academic degree	392 (71.2%)	188 (63.5%)	580 (68.5%)	0.060*
Health insurance coverage				
Yes	438 (79.5%)	205 (69.3%)	643 (75.9%)	
No	113 (20.5%)	91 (30.7%)	204 (24.1%)	0.001***
Birth place				
Urban	458 (83.1%)	218 (73.6%)	679 (79.8%)	
Rural	93 (16.9%)	78 (26.4%)	171 (20.2%)	0.001***
Monthly income US$				
Less than US$ 78	263 (47.7%)	158 (53.4%)	421 (49.7%)	
US$ 78–155	125 (22.7%)	72 (24.3%)	197 (23.3%0	
US$ 156–310	113 (20.5%)	52 (17.6%)	165 (19.5%)	
More than US$ 310	50 (9.1%)	14 (4.7%)	64 (7.6%)	0.008***
Own chronic (long-term) disease				
Yes	101 (18.3%)	58 (19.6%)	159 (18.8%)	
No	450 (81.7%)	238 (80.4%)	688 (81.2%)	0.653
Family member with chronic diseases such as cancer				
Yes	118 (21.4%)	68 (23.0%)	186 (22.0%)	
No	433 (78.6%)	228 (77.0%)	661 (78.0%)	0.602
Family member died in last year				
Yes	82 (14.9%)	47 (15.9%)	129 (15.2%)	
No	469 (85.1%)	249 (84.1%)	718 (84.8%)	0.700

*SD* standard deviation , *p < 0.1 , **p < 0.05 , ***p < 0.01

### The pattern of WTP responses

**Fig. 1:**
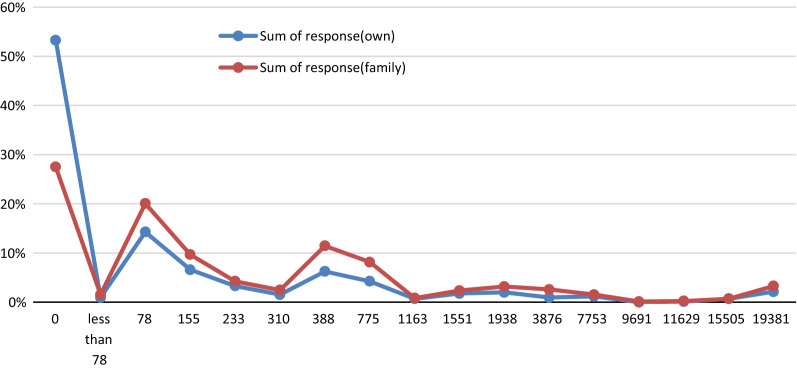
The rate of responses on each bid value for oneself and for a family member. The less than US$ 78, includes all WTP responses which respondents had positive WTP but indicated less than US$ 78

The findings showed a higher mean WTP value for a family member (US$ 1,355 ± SD 3,993) than for the individual participant (US$ 862 ± SD 3,224). About 65% of the individual participants had a WTP response (WTP > 0) for their own, and the payment rate increased to more than 90% WTP > 0 if a family member faced the risk of death (Fig. 1). Around 53% of the participants would have a WTP value of zero if they would encounter a life-threatening condition, and 28% would have a WTP value of zero if a family member would faced a life-threatening situation. In the mid-range of the bid values, the tendency towards paying for the family members was higher, but in the upper bid values, the participants showed similar behavior.

**Fig. 2:**
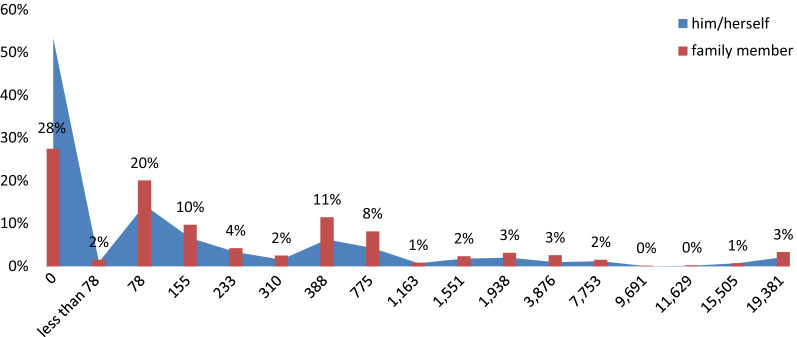
The stated WTP amount distribution of oneself and a family member. The less than US$ 78, includes all WTP responses which respondents had positive WTP but indicated less than US$ 78

Despite a higher tendency to pay for a family member to save a life, there was not a significant difference in the WTP pattern. The findings indicated that 75% of the respondents had the WTP value of less than US$ 155 for themselves, and 59% had the same WTP value as their family members. Only 2% of the participants had the WTP value of as high as US$19,381 (Fig. 2).

### WTP and WTP for additional QALY gained

The annual WTP value for the hypothetical life-saving treatment ranged from US$ 0 to 19,381 for the individual participants and US$ 0 to 38,763 for their family members. There was a slight difference between the mean utility value obtained using the EQ-5D-3L method and the VAS method (0.779 vs. 0.800). The WTP value for one additional QALY gained using the EQ-5D-3L method was US$ 1,202, while using the VAS method was US$1,101 (Table 2).

**Table 2:** Additional QALYs, WTP values and WTP per QALY values

WTP	Average ± SD	Minimum to maximum
	N = 847	
For oneself		
WTP per year ($US)	862 ± 3,224	0–19,381
Utility value using EQ-5D-3L	0.779 ± 0.168	0.10–0.89
Utility value using VAS	0.800 ± 0.204	0.11–1
WTP ($US) per QALY using EQ-5D-3L	1,202 ± 4,991	0–63,819
WTP ($US) per QALY using VAS	1,101 ± 4,143	0–42,640
For a family member		
WTP per year ($US)	1,355 ± 3,993	0–38,763
WTP per QALY	1,355 ± 3,993	0–38,763

### Factors affecting WTP per QALY values

The Tobit regression models indicated that the educational level, gender, birthplace, and monthly income were statistically significantly associated (p < 0.05) with the WTP for the additional QALY gained using both the EQ-5D-3L and the VAS methods (Table 3). Further, educational level and monthly income showed a statistically significant relationship with the WTP for the additional QALY gained by the family members (p < 0.05). The results of the marginal effects of the factors influencing the WTP revealed that females had a 9.3% and 8.2% higher probability of a WTP > $0 for the additional QALY gained from the life-saving treatment using the EQ-5D-3L and VAS methods, respectively than their male counterparts (Table 4). The WTP value of females for the additional QALY gained was about US$ 515 and US$ 388 more than the WTP of males using the EQ-5D-3L and the VAS methods, respectively.

**Table 3:** Results of the Tobit regression analysis of the factors affecting on WTP per QALY values

Explanatory variables	Model A	Model B	Model C
	β Coefficient	β Coefficient	β Coefficient
Age, year	− 45.0	− 39.4	− 1.3
Sex (ref. male)			
Female	1510.5*	1126.4*	477.7*
Marital status (ref. single)			
Married	335.4	226.5	572.4
Others	1717.6	479.8	1307.1
Education status (ref. academic degree)			
Illiterate	3351.5*	2162.5*	659.3
Primary and secondary school	− 260.8	− 497.9	− 812.9*
Health insurance coverage (ref. No.)			
Yes	842.9	992.6	− 16.1
Birth place (ref. rural)			
Urban	1398.4*	985.1*	488.2
Monthly income US$ (ref. less than 78)			
US$ 78 – 155	1060.5	506.4	338.9
US$ 156–310	1849.9*	1064.3*	771.7*
More than US$ 310	2864.8*	2094.6*	1388.0*
Having chronic disease (ref. no.)			
Yes	71.9	418.1	37.1
Family member with chronic diseases such as cancer (ref.no.)			
Yes	670.5	348.3	399.8
Family member died in the last year (ref.no.)			
Yes	563.0	505.8	595.7
LR chi2 [14]	46.9	38.0	31.0
Prob > chi2	< 0.001	< 0.001	0.005
Left-censored observations	296	296	80
Uncensored observations	551	551	767
Log likelihood	− 5785.2	− 5691.8	− 7543.4

*Model A* Dependent variable is WTP per QALY using EQ-5D-3L, *Model B* Dependent variable is WTP per QALY using VAS, *Model C* Dependent variable is WTP per QALY for family member

*Significance at p < 0.05

**Table 4:** Marginal effects of factors affecting on WTP per QALY values

Explanatory variables	Model A		Model B		Model C	
	Pr	E	Pr	E	Pr	E
Age, year	− 0.003	− 15.5	0.003	− 13.7	− 0.000	− 0.5
Sex (ref. male)						
Female	0.093*	515.1*	0.082*	388.3*	0.043	202.4
Marital status (ref. single)						
Married	0.021	114.6	0.016	78.5	0.052	242.8
Others	0.106	626.9	0.035	168.7	0.116	583.7
Education status (ref. academic degree)						
Illiterate	0.204*	1334.9*	0.158*	848.5*	0.058	299.8
Primary and secondary school	− 0.016	− 87.2	− 0.036	− 167.8	− 0.071*	− 333.9*
Health insurance coverage (ref. No.)						
Yes	0.051	289.4	0.073	344.4	− 0.001	− 6.85
Birth place (ref. rural)						
Urban	0.085*	460.7*	0.072*	330.1*	0.044	203.2
Monthly income US$ (ref. less than 78)						
US$ 78 – 155	0.064	354.1	0.037	171.1	0.031	140.7
US$ 156–310	0.113*	641.9*	0.078*	371.5*	0.070*	330.4*
More than US$ 310	0.175*	1044.6*	0.153*	775.9*	0.123*	620.4*
Having chronic disease (ref. no.)						
Yes	0.004	24.7	0.030	145.1	− 0.003	15.8
Family member with chronic diseases such as cancer (ref.no.)						
Yes	0.041	230.2	0.026	120.8	0.036	169.9
Family member died in the last year (ref.no.)						
Yes	0.034	193.3	0.037	175.5	0.054	253.2

*Model A* Dependent variable is WTP per QALY using EQ-5D-3L, *Model B* Dependent variable is WTP per QALY using VAS, *Model C* Dependent variable is WTP per QALY for family member, Pr shows the marginal effects for the probability of being uncensored and E indicates the marginal effects for the expected WTP per QALY value conditional on being uncensored: E (WTP per QALY | WTP per QALY > 0)

*Significance at p < 0.05

The findings of the marginal effect analysis using the EQ-5D-3L method revealed that the participants with moderate-income (US$ 156–310) and those with high income (more than US$ 310) respectively had a 11.3 and 17.5% higher probability of a WTP > $0 for the additional QALY gained from the life-saving treatment than those with the low income (less than US$ 78). Moreover, the participants with moderate-income and high-income had a US$ 641 and US$ 1,044 higher WTP for the additional QALY gained from the life-saving treatment, respectively, than those with low income.

## Discussion

Our findings indicated that the participants’ WTP values of US$ 1,100 using the VAS method and US$ 1,200 using the EQ-5D-3L method for the extra QALY gained from the hypothetical life-saving treatment. These values accounted for 0.20–0.24 of Iran’s GDP per capita in 2019 (US$ 5,506) and are far lower than the WHO suggested CE threshold value of one GDP per capita. The amount that the participants were willing to pay for the additional QALY gained from the life-saving treatment for a family member was about US$ 1,355, which represent 0.27 of the GDP per capita. These values are slightly lower than the WTP values of 0.22 to 0.56, and 0.57 of the GDP per capita for the additional QALY gained findings reported from studies in Iran [2, 30]. Comparatively speaking other countries such as Thailand found the WTP value of 1.42 times the GDP per capita for the additional QALY gained from life-saving interventions [4]. The strong positive relationship of the WTP for the extra QALY gained with the socioeconomic status of the participants might explain the difference.

The lower WTP for the additional QALY gained from the life-saving treatment observed in our study may be due to the declining trend of Iran’s GDP per capita in the last few years. For example, the GDP per capita decreased from US$ 7,818 in 2011 to US$ 5,506 in 2019 [31]. The GDP per capita of Iran (US$ 5,506) during the current study was also markedly lower than that of Thailand (US$ 7,500) in the year 2014, implying that increasing the GDP per capita may contribute to the increase in the CE thresholds [12].

Our findings highlight that the WHO CE threshold may be unrealistic for use by health policymakers to rationally choose cost-effective interventions in low- and middle-income countries (LMICs) like Iran, where the resources are limited. Others also point out a similar concern and reflect that the WHO CE threshold for choosing a cost-effective treatment is substantially high for LMICs [12]. Another possible reason for the differences in the CE threshold between ours and others might be due to the differences in concepts and methods used for eliciting the WTP for the additional QALY gained. For example, our study depended on the WHO's recommended CE threshold, which uses income [4], while others determined the CE threshold using opportunity costs [12] and preference [32]. The use of different scenarios such as life-saving treatments, life-prolonging treatment, and a difference in study population such as the use of the general population and a study on a specific disease can lead to differences in the WTP for the additional QALY gained. A study in Korea in the general population reported that the WTP for an additional QALY from a cure was more than twice (KRW 35 million vs. 15 million) that of the non-cure [33]. As well, the use of open-ended questions can be another reason for the difference in the WTP values for the extra QALY.

The VAS and EQ-5D-3L methods utilized in our study provided almost the same QALY values. Others from Thailand also reported closely related mean values for the additional QALY gained (0.872 vs. 0.853) using the VAS and the EQ-5D-3L methods [4]. The statistically significant difference in the WTP for the additional QALY for the individual participants and their family members observed in our study might relate to the importance of family. In a study conducted by Shiroiwa et al. showed that there was a higher QALY value for a family member compared to one’s value and indicated it may be because of altruistic utility and also the role of family in life [34]. Further, our study also showed that monthly income, educational level, and sex of the study participant were statistically significantly associated with the WTP for the extra QALY gained. Others also reported the positive association of income with the WTP value [4, 5, 13, 30], and higher income and educational level led to a higher WTP; and WTP more for the extra QALY gained [33].

## Strengths and limitations of the study

This study explored the WTP for the additional QALY gained from a hypothetical life-saving treatment and provided input for evidence-informed decisions in Iran. However, this study has some limitations, and the findings require cautious interpretations. First, the study depended on a sample taken from a general population in a specific geographic area. Hence, the observed CE threshold values cannot be generalizable to the entirety of Iran. Second, the study assumed a hypothetical family member as a healthy individual with a utility value of one, and the actual QALY valuation might not reflect this. Finally, the family member was not specified during the study period while there could be a wide variation ranging from a child to an elderly person. Thus, future studies need to consider these issues and related factors.

## Conclusions

The findings revealed that the values of the WTP for the additional QALY gained using hypothetical life-saving treatment varied from 0.20 to 0.24 of Iran’s GDP per capita. These values are far lower than the WHO recommended CE threshold of one GDP per capita, and there existed a strong relationship between the monthly income of the study participants and the WTP per capita values for the additional QALY gained. Future studies aiming to elicit the WTP values need to consider different scenarios of life-saving interventions to overcome the limitations observed in this study**.**

## Supplementary information

**Additional file 1.** English version of questionnaire

## Data Availability

The data used for the analysis in this study are available from the corresponding author upon reasonable request.

## Abbreviations

CE: Cost-effectiveness
GDP: Gross domestic product
LMICs: Low- and middle-income countries
OLS: Ordinary Least Square
QALY: Quality-adjusted life year
VAS: Visual analog scale
WTP: Willingness to pay
