# Supplementary material for: An exploratory study to estimate cost-effectiveness threshold value for life saving treatments in western Iran
Source: Cost Eff Resour Alloc. 2020 Oct 23;18:47. doi: 10.1186/s12962-020-00241-9 (PMC7585313; doi:10.1186/s12962-020-00241-9)
Supplement: Supplementary file 1 — Additional file 1. English version of questionnaire [file 12962_2020_241_MOESM1_ESM.docx]

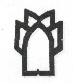
**Kermanshah University of Medical Sciences**

**Participant Information Sheet and Informed Consent Form**

My name is __________________. I am an academic staff of the Kermanshah University of Medical Sciences (KUMS). I kindly request you to lend me your attention to explain you about the study and to participate in the study.

**The research title:** *An explanatory Study to estimate cost-effectiveness threshold value for life saving treatments in Iran*

This study aims to elicit the monetary value of one living one additional year in a perfect health state from your perspective and the results of study will contribute to health policy decisions to improve healthcare.

**Aim of the study:** This study aims to elicit the willingness to pay of individuals for one additional quality-adjusted life-year gained from life-saving treatment and its related factors in Kermanshah city, the capital of Kermanshah province, western Iran.

**Procedure and duration:** I want to inform you that the study involves completing self-administered questionnaire to assess your health status, the sociodemographic characteristics and your preference for living one additional year in full health state using a life-saving treatment. It will take about 15 to 20 minutes. Hence, I kindly request you to spare me this time for the study.

**Risks and benefits:** This study is non-experimental and the risk of being participating in the study is minimal. However, it will take few minutes from your time. There will not be any direct payment, promotion or reward and even food to be served for participating in this study while the findings of the study are expected to be of paramount importance for the decision-makers to improve the health resource allocation in Iran.

**Confidentiality:** The information you provide will be confidential. Even there will no need of mentioning your name and related identifiers. The findings of the study will be generally focusing on purposive of the selected the study. No reference will be made in oral or written reports that can link participants to the information obtained.

**Rights:** The participation for the interview is fully voluntary. You have the right to declare to participate or not in this study. If you decide to participate, you have the right to withdraw from completing the questionnaire as well as from the interview process at any point you like without any explanation and this will not label you for any loss of the benefits that you otherwise are entitled in the community. If you decide to participate in the study, you have the right to ask questions for clarifications and get satisfactory answer. Besides, you do not have to answer any question that you do not want to answer.

**Contact address:** If there are any questions or enquires any time about the study, please contact:

- Dr Satar Rezaei: +98 …….

For any ethical related issues or concerns in the conduct of the study, please contact the ethics committee of the Deputy of Research, Kermanshah University of Medical Sciences chairperson:

- Dr. _____________ : +98……….

**Declaration of informed voluntary consent:**

I have read/it was read to me the participant information sheet. I have clearly understood the purpose of the study, the procedures, the risks and benefits, issues of confidentiality, the rights of participating and the contact address for any questions. I have been given the opportunity to ask questions for things that may have been unclear and get satisfactory answer. I was informed that I have the right to withdraw from the study at any time or not to answer any question that I do not want. Therefore, I declare my voluntary consent to participate in this study with my signature as indicated below. _________________________

**SECTION I: (EQ-5D-3L questionnaire)**

Please read the responses given under each of the following five dimensions of health state indicators carefully and put a tick mark on one of the boxes against each theme which best describes your own current health state.

1. **Mobility**

- I have no problem in walking about
- I have some problems in walking about
- I am confined to bed

1. **Self-Care**

- I have no problem with self-care
- I have some problems washing or dressing myself
- I am unable to wash or dress myself

1. **Usual Activities (e.g. work, study, housework, family or leisure activities)**

- I have no problem with performing my usual activities
- I have some problems with performing my usual activities
- I am unable to perform my usual activities

1. **Pain / Discomfort**

- I have no pain or discomfort
- I have moderate pain or discomfort
- I have extreme pain or discomfort

1. **Anxiety / Depression**

- I am not anxious or depressed
- I am moderately anxious or depressed
- I am extremely anxious or depressed

**VISUAL ANALOGUE SCALE (VAS) [Continue SECTION I)**

We have drawn a thermometer like scale which is numbered from 100 on the top, representing the best imaginable health state, to 0 at the bottom, showing the worst imaginable health state. Your current health state may be toward the best imaginable, around the middle, or toward the worst imaginable. Please think over where your current health state will fall on this scale, put a mark on the scale, and write the number shown on the scale reflecting your current health state in the three boxes given below against the scale.

Please draw a line on the scale to whichever point you feel indicates the death state of your imaginable current health state and write the number corresponding the mark on the scale on the below.

………….

Please draw a line on the scale to whichever point you feel indicates the perfect imaginable health state and write the number corresponding the mark on the scale on the space given below.

………….

Please draw a line on the scale to whichever point you feel indicates how good or bad your current health state is and write the number corresponding the mark on the scale on the space given below.

………….

**SECTION II (WTP Scenario) for the study participant**

“Suppose you had a life-threatening disease for one year. There is a treatment for your disease but if you do not take any treatment now, you will die today. If you get treated, you will be back to your current health state and live only for one more year”.

Please answer the following questions based on this scenario:

1. Are you willing to pay for receive the treatment? Yes No
2. If your response is **NO**; please stated the main reasons on the space provided below: …………………………………….
3. If your response is **YES**, what is the maximum amount of money which you are willing to pay *(in Iranian Rials)* to get the treatment? Please put a tick mark in one of the boxes given against the Card-1 shown below. ***Note: There is no compensation from health insurance organizations or government and you should pay it from your own pocket.***

**Card 1 (selected amount)**

 less than 10,000,000 IRR

 10,000,000 IRR

20,000,000IRR

 30,000,000 IRR

 40,000,000 IRR

 50,000,000 IRR

 100,000,000 IRR

 150,000,000 IRR

 200,000,000 IRR

 250,000,000 IRR

 500,000,000 IRR

 750,000,000 IRR

 1,000,000,000 IRR

 1,250,000,000 IRR

 1,500,000,000 IRR

 2,500,000,000 IRR

 > 2,500,000,000 IRR

**Card 2: If your maximum amount willingness to pay amount: is not indicated in the list given for Card-1 payment above, Please, state the exact amount below:**

………………… IRR

**SECTION II (WTP Scenario) for a family member**

“Suppose one of your family members had a life-threatening disease, and there is a treatment for it. If he/she does not take any treatment now, he/she will die today. If he/she gets, treated, he/she will back to his/her current health state and live only for one more year”.

Please answer the following questions based on this scenario:

1. Are you willing to pay for your family member to receive the treatment? Yes No
2. If your response is NO; please stated the main reasons for not willing to pay on the space provided: …………………………………….
3. If your response is YES, what is the maximum amount of money which you are willing to pay *in Iranian Rials for your family member* to get the treatment? Please put a tick mark in one of the boxes given against the Card-1 shown below. (***Note: There is no compensation from health insurance organizations or the government. The amount should be paid by your own).***

**Card-1 (selected amount)**

 less than 10,000,000 IRR

 10,000,000 IRR

 20,000,000IRR

 30,000,000 IRR

 40,000,000 IRR

 50,000,000 IRR

 100,000,000 IRR

 150,000,000 IRR

 200,000,000 IRR

 250,000,000 IRR

 500,000,000 IRR

 750,000,000 IRR

 1,000,000,000 IRR

 1,250,000,000 IRR

 1,500,000,000 IRR

 2,500,000,000 IRR

 > 2,500,000,000 IRR

**Card-2: If your maximum amount willingness to pay amount: is not indicated in the list given for Card-1 payment above, Please, state the exact amount below:**

**………………… IRR**

**SECTION III**

**Socioeconomic characteristics of the participant: Please write your response or put tick mark on the space provided as appropriate.**

1. Age (in years) ……………….
2. Marital status

Married Single Others

1. Birthplace

Urban Rural

1. Sex

Male Female

1. Educational status

Below high school High school and above

1. Health insurance coverage

Yes No

1. Monthly income in Iranian Rials (IRR)

Less than 10000000 IRR

Between 10000000 and 20000000 IRR

20000000 to 40000000 IRR

More than 40000000 IRR

1. Have own chronic (long-term) disease

Yes No

1. Had family member with cancer

Yes No

1. Family member died in last year

Yes No

**“Thank you for taking the time”.**
